# Carbon nanoparticle suspension could help get a more accurate nodal staging for patient with rectal cancer

**DOI:** 10.1038/s41598-021-89541-5

**Published:** 2021-05-11

**Authors:** Wei Ge, Qiang Li, Wen-jia Liu, Xiao-Qi Zhang, Xiang-shan Fan, Li-hua Shao, Liang Tao, Wen-xian Guan, Gang Chen

**Affiliations:** 1 Gastrointestinal Center, Nanjing Drum Tower Hospital, The Affiliated Hospital of Nanjing University Medical School, Nanjing, 210008 Jiangsu People’s Republic of China; 2 Department of Gastroenterology, Nanjing Drum Tower Hospital, The Affiliated Hospital of Nanjing University Medical School, Nanjing, 210008 Jiangsu People’s Republic of China; 3 Department of Pathology, Nanjing Drum Tower Hospital, The Affiliated Hospital of Nanjing University Medical School, Nanjing, 210008 Jiangsu People’s Republic of China

**Keywords:** Cancer, Gastroenterology

## Abstract

This study aimed to evaluate whether carbon nanoparticles could improve the accuracy of nodal staging in colorectal cancer (CRC). We performed a randomized controlled trial with CRC at the department of general surgery, the affiliated hospital of Nanjing University Medical School. A total of 160 patients were recruited in this research and 132 patients were included in the safety analyses. Among these patients, 72 cases were classified into control group and 60 cases into study group. The mean number of lymph nodes harvested from patients in study group was 19.3 ± 6.7 (range from 4 to 38), which was higher than that in control group (15.1 ± 5.7 (range from 3 to 29)) (p < 0.001). The mean number of positive lymph nodes got from patients in study group was 1.7 ± 3.5 (range from 0 to 22), which was also higher than that in control group (0.7 ± 1.4 (range from 0 to 7)) (p = 0.045). In study group, there were 30 patients (50%) proved to be N0, and remaining 30 patients (50%) were N1 or N2. However, 50 patients (69.4%) were N0 and 22 patients (30.6%) were N1 or N2 in control group. The rate of N0 in control group was significantly higher than that in study group (p = 0.023). Injecting carbon nanoparticle suspension could get a more accurate nodal staging to receive enough chemoradiotherapy, improving prognosis. Besides, injecting carbon nanoparticles suspension at four points 5 cm, 10 cm, 15 cm and 20 cm away from the anus by “sandwich” method was a new try.

**Trial registration**: This study was registered with ClinicalTrials.gov, number ChiCTR1900025127 on 12/8/2019.

## Introduction

Colorectal cancer (CRC) is one of the most common gastrointestinal malignancies in the world. Nowadays, the incidence of CRC is increasing every year, threatening the human health seriously. At present, the main treatment for CRC is radical resection. With the gradual development of CRC radical surgery technology, the resection rate and radical rate of CRC have significantly improved, and the death rate of patients also significantly decreased^1,2^. However, the 5-year survival rate of CRC is still low owing to the metastasis and recurrence following operation and the most common route of metastasis of CRC is lymph nodes. As a result, accurate assessment of lymph node metastasis and nodal staging in patients with CRC is very important^3,4^.

Currently, we cannot accurately determine intraoperative metastasis of regional lymph nodes in CRC. Therefore, in order to obtain more lymph nodes, some medical units carry out extensive lymph node dissection, leading to increased trauma and complication in patients. The National Comprehensive Cancer Network (NCCN) guidelines provide for at least 12 lymph nodes should be examined during a radical resection of CRC^5^. However, fewer than 12 lymph nodes were found in some cases, especially patients with rectal and sigmoid cancers. The reason may be that some lymph nodes are too small (< 5 mm) to be found or the mesentery is too thick to look for lymph nodes. The deficiency of lymph nodes harvested may affect lymph node staging, which may affect the subsequent chemotherapy further. For example, a single node that is positive moves patients from lymph node staging N0 to N1. The patients with lymph node staging N1 and N2 are generally treated the same, but N0 and N1 are treated completely differently. Lymph node staging N1 is a stronger indication for chemotherapy at many centers in contrast to N0. Therefore, enough lymph nodes harvested play a key role in improving prognosis and adjuvant treatment decision making. Tan et al. performed a study to investigate the prognostic impact of the number of lymph node dissected in rectal cancer patients after neoadjuvant therapy and they found that dissecting at least 12 lymph nodes may improve the patients’ overall survival, disease-free survival, and distant recurrence^6^. To harvest more lymph nodes, a special agent is needed to display the lymph nodes accurately for clinicians and pathologists to guide the range of lymph nodes dissection and obtain sufficient lymph nodes to assess the lymph node metastasis and guide the following treatments.

In the 1950s, methylene blue was used to trace lymph nodes. This method is simple and safe^7^, however, there are some limits, for example, the pigment will spread and fade with the passage of time and it must be observed within a limited time. Dye lymph node tracing has not been widely used. Then radionuclide markers were used in lymphatic imaging technology, but they required advanced equipment, professional technicians and necessary protections^8^. Recently, carbon nanoparticles tracer came into use, which is composed of nanoscale carbon particles with an average particle size of 150 nm. The nanoscale carbon particles are devoured by macrophages after being injected into the local tumor tissue under endoscopic or direct vision. Nanoscale carbon particles enter the lymphatic reflux system and stain the lymphatic tissue, then the other part continue to move to the next station to drain lymph nodes, and reached all levels of drainage lymph nodes in the region to form black stain^9,10^. Besides, it is simple to apply and has no harm to human body. At present, it has been accepted by most clinicians and is an ideal lymphatic tracer.

The aim of this study is to evaluate whether carbon nanoparticles could improve the accuracy of nodal staging in CRC.

## Materials and methods

### Study design and participants

We performed a randomized controlled trial between August, 2019 and December 2019. The inclusion criteria are (1) diagnosed as rectum or sigmoid colon adenocarcinoma by electronic colonoscopy and histopathology, (2) 18–75 years of age, (3) underwent radical resection, (4) the patients have good compliance and signed the informed consent. Exclusion criteria are (1) the patients have received neoadjuvant therapy before surgery, (2) carbon nanoparticles cannot be injected under colonoscopy, (3) acute intestinal obstruction or intestinal perforation, (4) combined with severe cardiopulmonary disease, (4) combined with severe coagulation mechanism disorder, (5) allergic to test drugs. Exit criteria are (1) serious adverse events occurred, (2) surgical treatment was not performed. Qualified patients are classified into study group and control group randomly. For enrolled patients, we collected age, gender, diagnosis, previous history, operations, and so on before surgery. This study was registered with ClinicalTrials.gov, number ChiCTR1900025127 on 12/8/2019. Written informed consents were provided by all the participating patients, and this study was approved by the IRB of Nanjing Drum Tower Hospital, the affiliated hospital of Nanjing University Medical School.

### Randomisation and masking

Patients were randomly assigned into study group and control group. Randomisation was done by an independent statistician using a computer-generated randomisation list. A computer-generated sequential randomisation list using variably sized permuted blocks was prepared by the trial statistician, and incorporated securely into the online trial database. The list was concealed until allocation by the prevention of access by any database users through login-based permissions; after eligibility was confirmed, researchers then did the randomization. At each site, the principal investigator enrolled participants and assigned patients to the trial groups. Investigators and outcome evaluators were blinded to treatment allocation. While endoscopists, surgeons and pathologists could not be blinded to treatment allocation. Because patients in study group underwent injecting carbon nanoparticles, so endoscopists were not blinded to them. The patients in the study group were known to have black stained lymph nodes during the operation and specimen processing, so, they were not blinded to surgeons and pathologists.

### Procedures

Participants assigned to the study group underwent electronic colonoscopy at least 1 day before surgery and 0.5 ml (25 mg) of carbon nanoparticles are injected into the submucosal layer. We diluted 0.5 ml of carbon nanoparticles to 1 ml. Submucosal injection of 0.25 ml carbon nanoparticles was performed at four points 5 cm, 10 cm, 15 cm and 20 cm away from the anus by sandwich method. The carbon nanoparticles are provided by Chongqing Lummy Pharmaceutical Co., Ltd. The patients of control group did not receive submucosal injection of carbon nanoparticles. All participants underwent radical operation by the same group of surgeons. The removal specimens were sent to pathology department and underwent pathological examination. The lymph nodes were manually harvested from fresh specimens as many as possible. The pathologic examination and nodal staging were taken by the same experienced pathologists.

### Outcomes

The primary endpoints were total number of lymph nodes, number of positive lymph nodes, and nodal staging. The secondary endpoints were number of “253” lymph nodes and number of positive “253” lymph nodes. The “253” lymph node is the D3 station lymph nodes for colorectal cancer, which lies from the origin of inferior mesenteric artery to the left colonic artery. The nodal staging is according to the guideline of Chinese Society of Clinical Oncology CSCO2018. Patients with no lymph node metastasis are regarded as N0, with one to three lymph nodes metastasis as N1, and with more than three lymph nodes metastasis as N2^11^.

### Statistical analysis

Data are presented as mean (± SD) for continue variables and as frequency (%) for categorical variables. For comparisons, we used two-tailed student *t* test to evaluate the continuous variables and the Chi-square test or Fisher’s exact test for categorical variables. *P* < 0.05 was considered statistically significant. All statistical calculations were performed using SPSS software (version 19.0).

### Ethics approval

This study was approved by the IRB of Nanjing Drum Tower Hospital, the affiliated hospital of Nanjing University Medical School.

### Consent to participate

We thank the patients for giving us written consent for publishing their details.

### Consent for publication

The publication of the paper has been approved by all authors.

## Results

### Clinical characteristics of the patients

**Figure 1:**
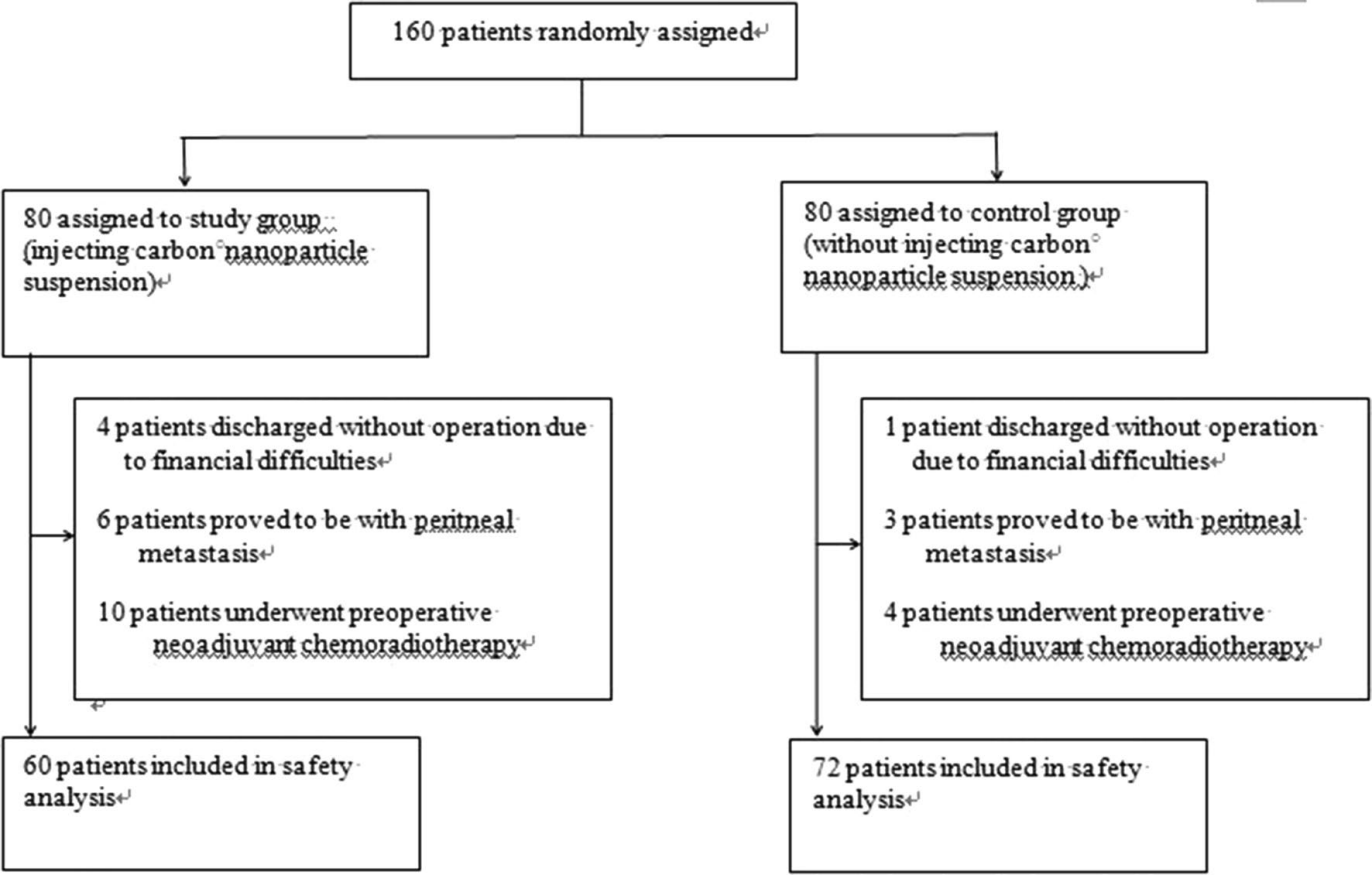
Trial profile.

A total of 160 patients were recruited in this research and randomly assigned: 80 to the study group and 80 to the control group (Fig. 1). In study group, 4 patients discharged without operation due to financial difficulties, 6 patients proved to be with peritoneal metastasis and 10 patients underwent preoperative neoadjuvant chemoradiotherapy as some enlarged mesenteric lymph nodes considering metastasis. These 20 patients in study group were excluded from the safety analysis. In study group, one patient discharged without operation due to financial difficulties and 3 patients proved to be with peritoneal metastasis and 4 patients underwent preoperative neoadjuvant chemoradiotherapy as some enlarged mesenteric lymph nodes considering metastasis. So, these 8 patients were also excluded from the safety analysis (Fig. 1). Hence, 132 patients were included in the safety analyses. Among these patients, 72 cases were classified into control group and 60 cases into study group. The control group included 42 men and 30 women, ages ranging from 32 to75 years, with mean age 64.2 years. Similarly, there were 30 men and 21 women in study group, ages ranging from 40 to 75 years, with mean age 61 years. The diagnoses of recruited patients were sigmoid cancer in 52 cases and rectal cancer in 80 cases. There were 24 cases with sigmoid cancer and 48 cases with rectal cancer in control group. The remaining 28 cases with sigmoid cancer and 32 cases with rectal cancer were classified into study group. Of all patients, a laparoscopic technique was used in 130 cases (98%). All patients underwent radical resection with D3 lymph node dissection. The patients’ clinicopathologic characteristics were shown in Table1 and there were no statistic differences between the two groups.

**Table 1:** The clinical characteristics of the patients stratified by group.

	Control group	Study group	t/Chi-square value	*p* value
Age mean years	64.2 ± 10.4	61.0 ± 11.7	1.722	0.087
**Gender**				
Male	42	39	0.614	0.433
Female	30	21		
**Diagnosis**				
Sigmoid cancer	24	28	2.437	0.119
Rectal cancer	48	32		
**Operation**				
Laparoscopy	71	59		
Laparotomy	1	1		
Tumor size (cm)	3.06 ± 1.41	2.81 ± 1.14	− 1.118	0.266
Length of colon removed (cm)	15.31 ± 0.99	15.6 ± 1.00	1.7	0.092
**T stage**			0.511	0.775
T1	35	30		
T2	33	25		
T3	4	5		
Operation time (min)	163 ± 27	169 ± 24	1.373	0.172
Amount of bleeding (ml)	88 ± 36	83 ± 34	− 0.924	0.357
Hospital stay (days)	7. ± 0.8	7.1 ± 0.7	− 1.698	0.092

### The comparison of lymph node acquisition between the two groups

**Figure 2:**
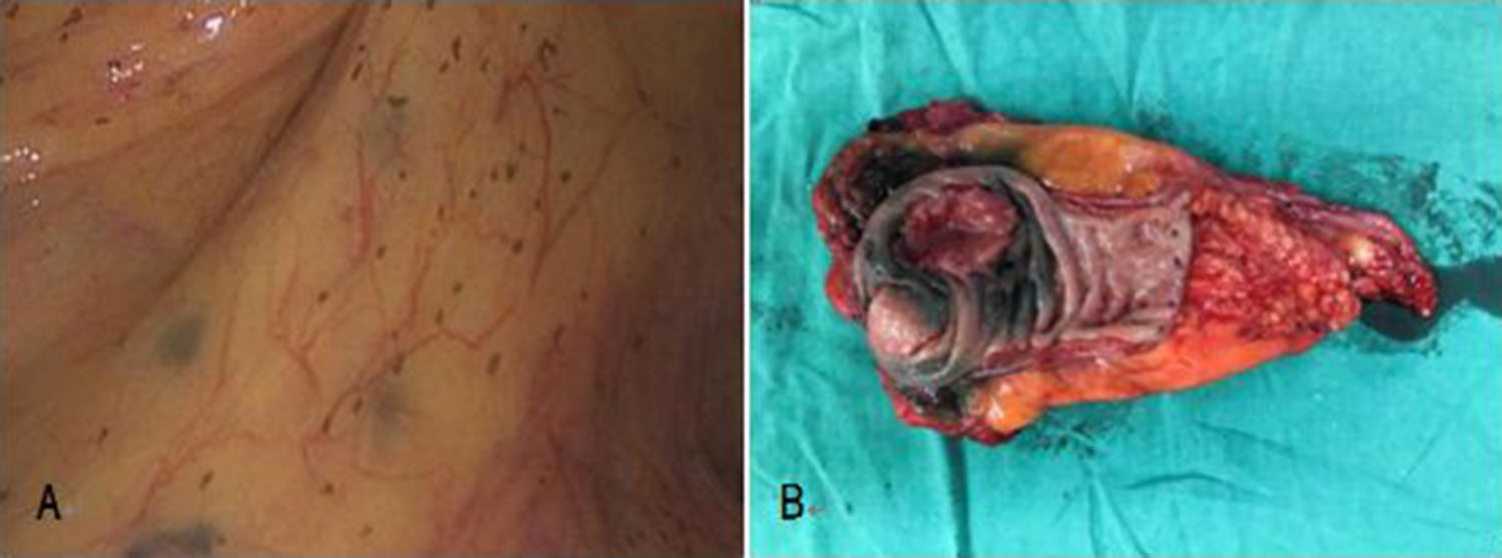
The lymph node was dyed black by carbon nanoparticle suspension during operation (A); we could get the lymph nodes from the specimen easily.

The lymph nodes can be traced well by carbon nanoparticle suspension as in Fig. 2. The mean number of lymph nodes harvested from patients in study group was 19.3 ± 6.7 (range from 4 to 38), which was higher than that in control group (15.1 ± 5.7 (range from 3 to 29)) (p < 0.001). The mean number of positive lymph nodes got from patients in study group was 1.7 ± 3.5 (range from 0 to 22), which was also higher than that in control group (0.7 ± 1.4 (range from 0 to 7)) (p = 0.045). Besides, we recorded the numbers of “253” lymph nodes separately. The result showed that the mean numbers of “253” lymph nodes was 2.9 ± 2.9 (range from 0 to 11) in study group and 2.2 ± 2.0 (range from 0 to 11) in control group without statistic difference (p = 0.153). There was only one positive “253” lymph node in study group and no positive “253” lymph node in control group (p = 0.323). All these details were summarized in Table 2.

**Table 2:** The comparison of lymph node acquisition between control and study groups.

	Control group	Study group	t value	*p* value
Total number of lymph nodes	15 ± 5.7	19 ± 6.6	− 3.863	0.000
Number of positive lymph nodes	0.7 ± 1.4	1.7 ± 3.5	− 2.037	0.045
Total number of 253 lymph nodes	2.2 ± 2.0	2.9 ± 2.9	− 1.442	0.153
Number of positive 253 lymph nodes	0.00 ± 0.00	0.02 ± 0.15	− 1.000	0.323

### Effect of carbon nanoparticle suspension on nodal staging

**Figure 3:**
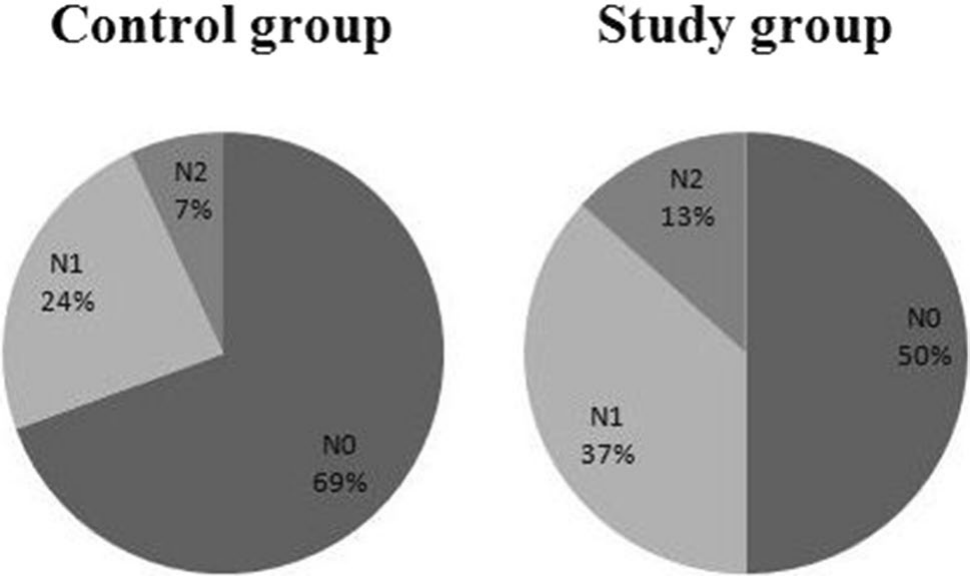
The constituent ratios of N0, N1, and N2 in study and control groups without statistic difference (*P* = 0.071).

In study group, there were 30 patients (50%) proved to be N0, and remaining 30 patients (50%) were N1 or N2. However, 50 patients (69.4%) were N0 and 22 patients (30.6%) were N1 or N2 in control group (Table 3). The rate of N0 in control group was significantly higher than that in study group (p = 0.023). We then drew a pie chart to show the constituent ratio of N0, N1, and N2 in the two groups in Fig. 3, showing no statistic difference (p = 0.071).

**Table 3:** The effect of carbon nanoparticle suspension on nodal staging.

	Control group	Study group	Chi-square value	p value
N0	50 (69.4%)	30 (50%)	5.183	0.023
N1/N2	22 (30.6%)	30 (50%)

## Discussion

At present, TNM stage plays an important role in making treatment strategies and prognostic judgement for CRC. TNM stage is mainly based on the depth of tissue infiltration, regional lymph node metastasis and distant metastasis. Lymph node metastasis is an important prognostic factor for CRC patients before distant metastasis presents, as lymph nodes metastasis determined the program and course of follow-up treatment. So the pathologists should find the lymph nodes as many as possible to avoid missing positive lymph nodes, especially small positive lymph nodes (< 5 mm). However, we cannot get enough lymph nodes (more than 12 lymph nodes) in some patients, especially patients with rectal cancer. These patients are classified as a cancer group with high risk factor and needed to receive chemo-radiotherapy according to the guidelines, even if the TNM stage is I. Among these, in our opinion, some patients undergo over-treatment owing to getting not enough lymph nodes. In addition, the deficiency of lymph nodes harvested may affect lymph node staging, which may affect the subsequent chemotherapy further. For example, a single node that is positive moves patients from lymph node staging N0 to N1 and is also a stronger indication for chemotherapy at many centers. Therefore, we should try our best to get more lymph nodes from the specimen.

Carbon nanoparticle suspension was used to trace lymph nodes since 2007, which gave us a new horizon to clear away and get lymph nodes. The carbon nanoparticle suspensions with average diameter of 150 nm only enter lymphatic vessel capillaries (diameter of 150–500 nm) rather than blood vessel capillaries (diameter of 20–50 nm). So, carbon nanoparticle suspension is a good lymph nodes tracer. There were studies showed that no abdominal pain, fever, diarrhea, and other symptoms of infection were found in patients with injecting carbon nanoparticle suspension, confirming the safety of carbon nanoparticle suspension^9^. In our study, we showed that patients with CRC injected of carbon nanoparticle suspension could get more lymph nodes, especially positive lymph nodes to improve the accuracy of nodal staging. Besides, the rate of patients with N1 or N2 injecting of carbon nanoparticle suspension was higher than that without injecting. So, we conclude that injecting carbon nanoparticle suspension could get a more accurate nodal staging to receive enough chemo-radiotherapy, improving prognosis.

In this research, we studied “253” lymph nodes separately and there were no statistic differences in the numbers of lymph nodes and positive lymph nodes between the two groups. Therefore, we concluded that injecting carbon nanoparticle suspension did not affect the range of “253” lymph node dissection. The reason may be that the “253” lymph nodes have an exact range and these lymph nodes are easy to be found by pathologists. Besides, of all 132 patients, there was only one patient (0.8%) with “253” lymph nodes metastasis. Therefore, further studies are needed to determine whether “253” lymph nodes dissection is required in all patients with CRC.

Our study mainly put emphasis on patients with sigmoid and rectal cancers, as it was difficult to get enough lymph nodes in these patients sometimes. There were also a few similar studies. Zhang et al. analyzed the effect of injection of carbon nanoparticle suspension on the outcomes of patients with mid-low rectal cancer. Their results showed that the mean number of lymph nodes removed in the experimental group was higher than that in control group^10^, which was consistent with our results. However, the positive lymph nodes and nodal staging were not compared in this study, which were more important and valuable. Wang et al. evaluated the effect of carbon nanoparticles in improving lymph node detection and staging accuracy in patients with rectal cancer who received neoadjuvant chemo-radiotherapy followed by curative resection. They found that the mean number of lymph nodes retrieved in the experimental group was more than that in the control group (p < 0.001) and the percentage of patients with positive lymph nodes was lower than in the control group (p = 0.037)^12^. However, they did not compare the constituent ratios of N0, N1, and N2 between the two groups respectively, which was done in our study with no statistic difference.

There were also other studies on colon cancer of other sites, such as right hemicolon. Pan et al. performed a study to analyze and evaluate the feasibility of using carbon nanoparticles to track lymph nodes metastases in right colon tumors. Their results showed that the number of total harvested lymph nodes and the number of positive patients in the experimental group increased significantly compared with the control group. Besides, the increase of positive percentage shifted some patients toward high stage, although the total number of positive lymph nodes changed a little bit^13^. However, the researches on right hemicolon were rare. The reason may be that the mesentery of right hemicolon was so thin that it was easy to harvest lymph nodes. To discover the positive lymph nodes less than 2 mm, we should also expand the study of the right colon.

The methods of injecting carbon nanoparticle suspension were various. According to the specification, the injection was performed at 4–6 points around the tumor under endoscope or through the anus. Most studies referred to the instructions for injecting carbon nanoparticles. In our study, we adopted a new method that we diluted 0.5 ml of carbon nanoparticles to 1 ml firstly and then injected 0.25 ml carbon nanoparticles suspension at four points 5 cm, 10 cm, 15 cm and 20 cm away from the anus. To reduce the waste of nanocarbon, we filled saline at the two ends of the carbon nanoparticles in the injection device and we named this method as “sandwich”. The advantage of this method was that the carbon nanoparticle suspension could be dispersed more evenly in the mesocolon and dyed more lymph nodes. As far as we know, this was the first study to inject carbon nanoparticle suspension by this method.

Up to now, carbon nanoparticle suspension is used to trace lymph nodes not only in CRC, but also in breast cancer, thyroid cancer, gastric cancer and so on. Most of axillary lymph nodes were stained black by the suspension of carbon nanoparticles, which helped identify the lymph nodes from the surrounding tissue and avoided aggressive axillary treatment for breast cancer^14^. There were studies showed that carbon nanoparticle lymph node tracer improved the outcomes of surgical treatment in papillary thyroid cancer^15^. Besides, it was feasible and satisfactory to use carbon nanoparticle to show sentinel lymph nodes in early gastric cancer to choose proper operation^16,17^. In my opinion, carbon nanoparticle suspension could be used in any malignant tumors, which needed lymph nodes cleaning.

## Conclusion

Submucosal injection of carbon nanoparticle suspension could get more lymph nodes, especially positive lymph nodes for patients with CRC. The rate of patients with N1 or N2 injecting of carbon nanoparticle suspension was higher than that without injecting. So injecting carbon nanoparticle suspension could get a more accurate nodal staging to receive enough chemoradiotherapy, improving prognosis. Besides, injecting carbon nanoparticles suspension at four points 5 cm, 10 cm, 15 cm and 20 cm away from the anus by “sandwich” method was a new try.

### Prospect

This randomized clinical trial got a very valuable conclusion that injecting carbon nanoparticle suspension got more positive lymph nodes and improved the rate of N1 or N2 for patients with CRC. This also indirectly suggested that there were positive lymph nodes not been found in some patients without injecting carbon nanoparticle suspension. In order to get a more accurate lymph node stage for patients with CRC, we suggest that each patient should be injected carbon nanoparticle suspension before surgery. Of course, this conclusion should be confirmed further owing to the limit of the simple size.
